# Controlled Growth of Rubrene Nanowires by Eutectic Melt Crystallization

**DOI:** 10.1038/srep23108

**Published:** 2016-03-15

**Authors:** Jeyon Chung, Jinho Hyon, Kyung-Sun Park, Boram Cho, Jangmi Baek, Jueun Kim, Sang Uck Lee, Myung Mo Sung, Youngjong Kang

**Affiliations:** 1 Department of Chemistry, Research Institute for Natural Sciences, and Institute of Nano Science and Technology, Hanyang University, 222 Wangsimni-Ro, Seongdong-Gu, Seoul, 04763 (Korea); 2 Department of Chemistry and Applied Chemistry, Hanyang University, 55 Hanyangdaehak-ro, Sangnok-gu, Ansan, Gyeonggi-do, 15588 (Korea)

## Abstract

Organic semiconductors including rubrene, Alq_3_, copper phthalocyanine and pentacene are crystallized by the eutectic melt crystallization. Those organic semiconductors form good eutectic systems with the various volatile crystallizable additives such as benzoic acid, salicylic acid, naphthalene and 1,3,5-trichlorobenzene. Due to the formation of the eutectic system, organic semiconductors having originally high melting point (*T*_m_ > 300 °C) are melted and crystallized at low temperature (*T*_e_ = 40.8–133 °C). The volatile crystallizable additives are easily removed by sublimation. For a model system using rubrene, single crystalline rubrene nanowires are prepared by the eutectic melt crystallization and the eutectic-melt-assisted nanoimpinting (EMAN) technique. It is demonstrated that crystal structure and the growth direction of rubrene can be controlled by using different volatile crystallizable additives. The field effect mobility of rubrene nanowires prepared using several different crystallizable additives are measured and compared.

Eutectic mixtures, which are solid mixtures melting and solidifying at lower temperature than any of pure ingredients, have been widely utilized in metallurgy to cast engineering alloys for a long time^1^,^2^,^3^. Melting-point depression in the eutectic system allows to process solids with high melting point at moderately low temperature. Tin and lead eutecic alloy for soldering is a good example^4^. Melting-point depression in eutectic system was also actively used in organic mixtures as well as in metallurgy. Recently, it was reported that deep eutectic solvensts consisting of two organic solid precursors yield free flowing fluids at room temperature, and which were utilized as alternative solvents of ionic liquids^5^,^6^,^7^,^8^. Besides melting-point depression, eutectic systems can be used to produce the micro- or nanostructures by using the eutectic reaction, a phase transition between liquid and mixture of solid phases (Fig. 1a). When a molten eutectic mixture is cooled down below the eutectic point, the eutectic reaction causes various morphologies including lamellae, cylinders, spheres and inter-connected structures^1^,^2^. During the eutectic reaction, the two components concurrently crystallize and epitaxial growth was frequently observed at the binary interfaces^9^,^10^,^11^,^12^,^13^,^14^. For example, well-ordered block copolymer microdomain patterns were formed by epitaxy during the eutectic reaction between block copolymers and a crystallizable additive^10^,^11^,^12^.

Organic semiconductors based on conjugated small molecules or polymers have been extensively investigated as promising materials for optical and electronic devices^15^,^16^,^17^,^18^,^19^,^20^,^21^. A multitude of applications including sensors^22^,^23^,^24^,^25^, phototransistors^26^,^27^,^28^, solar cells^29^,^30^, memory devices^31^,^32^, and organic field-effect transistors^33^,^34^ have been demonstrated using organic semiconductors. Since optical and electrical properties of organic semiconductors are highly dependent on their crystallinity and crystal structure as well as intrinsic molecular structure, there have been many research efforts to grow organic semiconductor crystals with controlled crystal structure^15^,^16^,^35^,^36^. While high vacuum deposition and solution casting techniques have been adopted to this end, other alternative methods applicable to the molecules that can not be grown to crystal by using the conventional methods because of their low vapor pressure or poor solubility are still highly demanded. Melt crystallization is a versatile tool for purifying and growing organic and inorganic materials, but it has been barely explored for the crystallization of organic semiconductors because of their high melting point which is close to or even higher than the decomposition tempearture.

Herein, we report a melt crystallization of organic semiconductors assisted by eutectic crystallization. Our system utilizes the binary mixtures consisting of an organic semiconductor and a volatile crystallizable additive (VCA). Due to the strong melting-point depression in the entectic system, organic semiconductors with high melting-point can be melted and crystallized at moderately low temperature (*T*_e_ = 40.8–133 °C). The eutectic reaction between organic semiconductor and VCA was utilized to control the morphology and the crystal structure of organic semiconductor. In our experiments, the initial composition of the mixture was intentionally brought to the hypereutectic regime where the content of VCA is larger than that of organic semiconductor to create well-defined nanowire structure. It is worthwhile mentioning that our target organic semiconductor however crystallizes only at the eutectic point (Fig. 1a) although the initial composition is away from the eutectic point. As cooling the molten liquid, the abundant VCA first precipates, and the composition of the molten liquid is getting close to the eutectic point following the liquidus line. Finally, semiconductor crystallizes simultaneously with VCA at the eutectic composition as the temperature is lowered below the eutectic temperature (*T*_e_)^3^. Such concurrent crystallization at the eutectic composition is a key for controlling the crystal structure of semiconductor by using various VCAs. It was found that the crystal structure of the resulting organic semiconductor is highly dependent on the species of VCA. Choosing appropriate VCA is crucial for the successful crystal growth by eutectic melt crystallization. In our experiments, crystalline solids with moderately high vapor pressure such as benzoic acid (BA), naphthalene (NAP), salicylic acid (SA) and 1,3,5-trichlorobenzene (TCB) were used for VCA materials. Due to their high vapor pressure, the VCA can be easily removed by sublimation, and pure organic semiconducting nanowires can be isolated without destruction of the fabricated structure. As demonstration, various organic semiconducting nanowires were fabricated by using the eutectic melt crystallization of rubrene, pentacene, Alq_3_, and copper phthalocyanine.

## Results and Discussion

### Preparation of rubrene nanowires by eutectic melt crystallization using BA (RB-NW_BA_)

**Figure 1:**
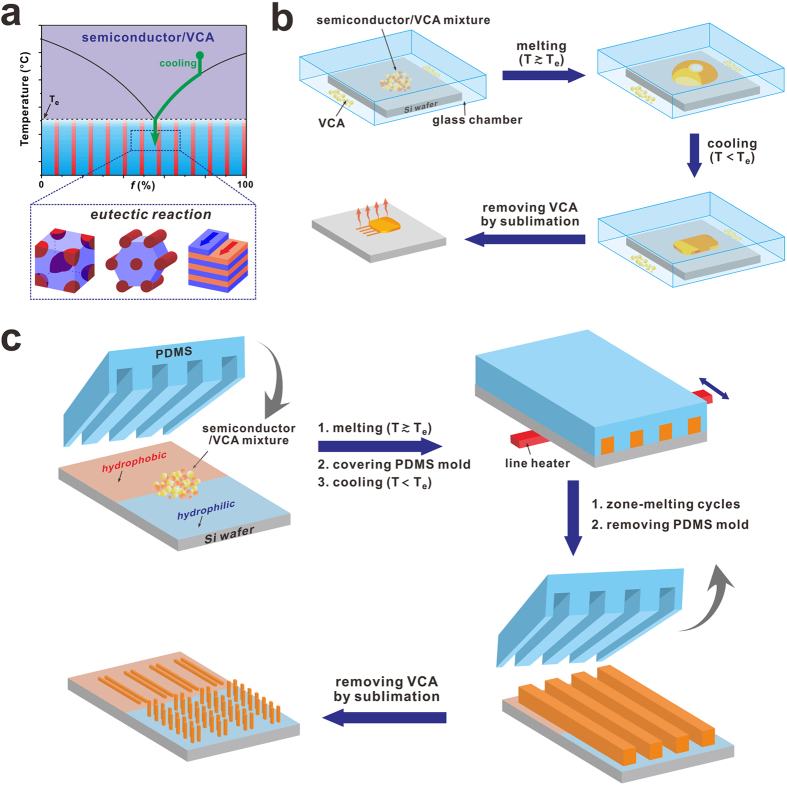
Schematic illustrations showing the preparation method of rubrene nanowires by eutectic melt crystallization.

Organic semiconducting nanowires were prepared by sequential three step processes, eutectic melting, crystallization and removal of VCA by sublimation (Fig. 1b). For example, rubrene nanowires were prepared by eutectic melt crystallization using BA as a VCA. To set the temperature profile, the phase diagram of binary mixture of rubrene and BA was first investigated as a function of the weight fraction of benzoic acid (*f*_BA_) (Figure S1a). For eutectic melt crystallization, well-ground powder mixture of rubrene and BA with a certain composition (0.3 ≤ *f*_BA_ ≤ 0.98) was first melted on a silicon wafer at 130 °C which is slightly higher than the eutectic temperature (*T*_e_ = 119 °C). During the eutectic melting process, the samples were placed in a small chamber which is pre-saturated with BA vapor to prevent the composition change of the mixtures. After complete melting, temperature was lowered to 100 °C with controlled cooling rate (*v*_cooling_ = −2 ~ −15 °C/min), and then cooled down to the room temperature. To prevent thermal oxidation, melting and crystallization processes were carried out under argon atmosphere. Finally, rubrene nanowires (RB-NW_BA_) were isolated by sublimating BA under the mild vacuum condition (1 torr) at 50 °C for 24 hr. To characterize the stability of rubrene during the eutectic melt crystallization, the contents of rubrene oxide (C_42_H_28_O) and rubrene peroxide (C_42_H_28_O_2_) were analyzed by means of MALDI-TOF spectrometry (Figure S2). The as-purchased rubrene powder was slightly oxidized exhibiting rubrene oxide peak at m/z = 548.9 (1.1%) and rubrene peroxide peak at m/z = 564.9 (2.6%) (Figure S2a). Due to the relatively low activation energy of rubrene peroxide for thermal decomposition (*E*_a_ = 30.4 kcal/mol)^37^, rubrene can be regenerated by thermal annealing. As shown in Figure S2b, the content of rubrene peroxide (2.6%) was slightly decreased to 1.3% after thermal annealing at 130 °C, while the rubrene oxide content was unchanged. Interestingly, the contents of oxidized rubrene were much less after eutectic melt crystallization (0.1% of rubrene oxide and 0.21% of rubrene peroxide) (Figure S2c).

**Figure 2:**
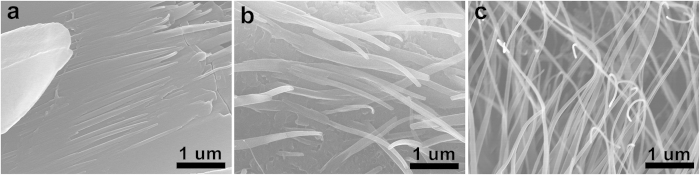
Morphological evolution of rubrene by eutectic melt crystallization depending on the composition: (**a**) *f*_BA_ = 0.6, (**b**) *f*_BA_ = 0.8, and (**c**) *f*_BA_ = 0.9. The temperature was abruptly cooled from 130 °C to 25 °C.

**Figure 3:**
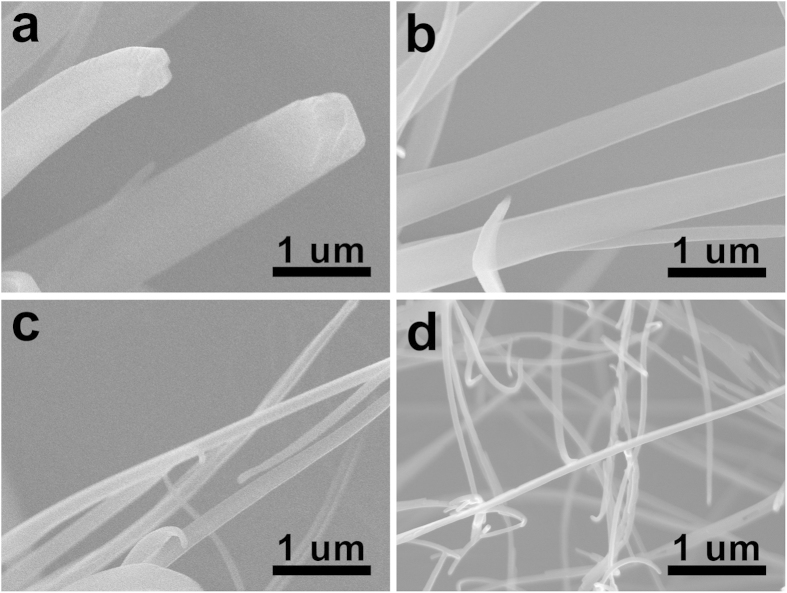
Kinetic control of the width of RB-NW_BA_ as a function of cooling rate (*v*_cooling_) in a strong hypereutectic regime (*f*_BA_ = 0.9); (**a**) *W* = 755 ± 90 nm at *v*_cooling_ = −5 °C/min, (**b**) *W* = 358 ± 48 nm at *v*_cooling_ = −10 °C/min, (**c**) *W* = 199 ± 42 nm at *v*_cooling_ = −15 °C/min, (**d**) *W* = 78 ± 25 nm when the temperature was abruptly dropped from 130 °C to 25 °C.

The morphology of rubrene was dependent on both composition and cooling rate. At the same experimental condition, plate-like rubrene was originally observed at low *f*_BA_, and then wire-like structures were gradually developed as increasing *f*_BA_. As shown in Fig. 2a, rubrene platelets were dominantly observed and small fractions of wires were only observed at the edge of the plates at *f*_BA_ = 0.6. The short wires were gradually evolved into the long isolated wires as *f*_BA_ increased to 0.8 (Fig. 2b), and fully isolated wires became dominant at the strong hypereutectic regime (*f*_BA_ ≥ 0.9) (Fig. 2c). In this case, it is noteworthy that the crystallization of rubrene occurs only at the eutectic composition regardless of the initial composition. To optimize the condition for making rubrene nanowires, the effect of the cooling rate on the morphology of rubrene was further investigated at the strong hypereutectic regime (*f*_BA_ = 0.9) (Fig. 3). Relatively thick rubrene wires (*W* = 755 ± 90 nm) were obtained at the slow temperature gradient (*v*_cooling_ = −5 °C/min). With increasing the cooling rate, the width gradually decreased; for example *W* = 358 ± 48 nm at *v*_cooling_ = −10 °C/min and *W* = 199 ± 42 nm at *v*_cooling_ = −15 °C/min. The thinnest rubrene nanowires were obtained when the temperature abruptly dropped from 130 °C to 25 °C (*W* = 78 ± 25 nm). The average length of RB-NW_BA_ was typically on the order of several hundred micrometers. The cooling rate dependent size of RB-NW_BA_ is consistent with typical behaviors of bulk crystals where the grain size is highly dependent on the temperature profile of crystallization^38^,^39^.

**Figure 4:**
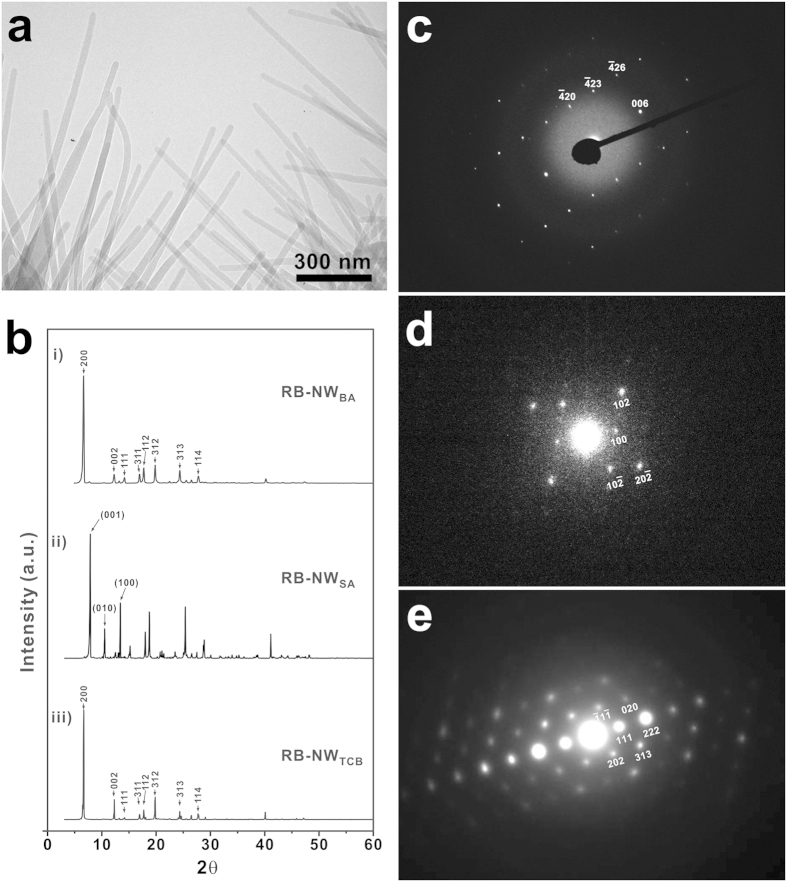
(**a**) A representative TEM micrograph of RB-NW_BA_ prepared by eutectic melt crystallization on a several-gram scale. (**b**) XRD patterns of rubrene nanowires prepared by eutectic melt crystallization: i) RB-NW_BA_, ii) RB-NW_SA_, and iii) RB-NW_TCB_. The XRD patterns of RB-NW_BA_ and RB-NW_TCB_ were both consistent with the orthormbic structure (*a* = 26.86, *b* = 7.193, *c* = 14.433, *α* = *β* = *γ* = 90), and RB-NW_SA_ was consistent with the triclinic structure (*a* = 7.02, *b* = 8.54, *c* = 11.95, *α* = 93.04, *β* = 105.58, *γ* = 96.28). SAED patterns of (**c**) RB-NW_BA_, (**d**) RB-NW_SA_, and (**e**) RB-NW_TCB_.

Eutectic melt crystallization can be easily scaled up. For XRD measurements, a few grams of RB-NW_BA_ were prepared by doing the eutectic melt crystallization in a glass vial. TEM analysis reveals that the morphological structure of the RB-NW_BA_ synthesized in a large scale is same as that synthesized in a small scale (Fig. 4a). XRD pattern of the RB-NW_BA_ shows narrow and well defined peaks, which corresponds to the simulated XRD pattern of orthorhombic structure with lattice parameter of *a* = 26.86, *b* = 7.193, *c* = 14.433, *α = β = γ* = 90 (Fig. 4b–i)^40^. Furthermore, the RB-NW_BA_ are single-crystalline as imparted by the sharp spots in the corresponding SAED patterns (Fig. 4c). The SAED patterns obtained from two different regions in a nanowire were identical. Based on the crystal information obtained by XRD and SAED analysis, it was determined that the RB-NW_BA_ preferentially grow along [001] direction. To understand the crystal growth of rubrene on BA, the interface energy between BA and rubrene was calculated^41^,^42^. The favorable facet and growth direction of rubrene on the (002) BA were determined by calculating both the interaction energy and the cell distortion energy (Table 1)^43^. Considering both interaction energy and cell distortion energy between rubrene surfaces and (002) benzoic acid, it was expected that rubrene preferentially grows along [001] direction with facing the plane (001) on the (002) of benzoic acid. In this case, the *c*-axis of rubrene is parallel with the *b*-axis of benzoic acid (Figure S3). These calculation results are well consistent with the crystal structure of rubrene nanowires determined by XRD and SAED.

### Vertical-lateral growth control of RB-NW_BA_

**Figure 5:**
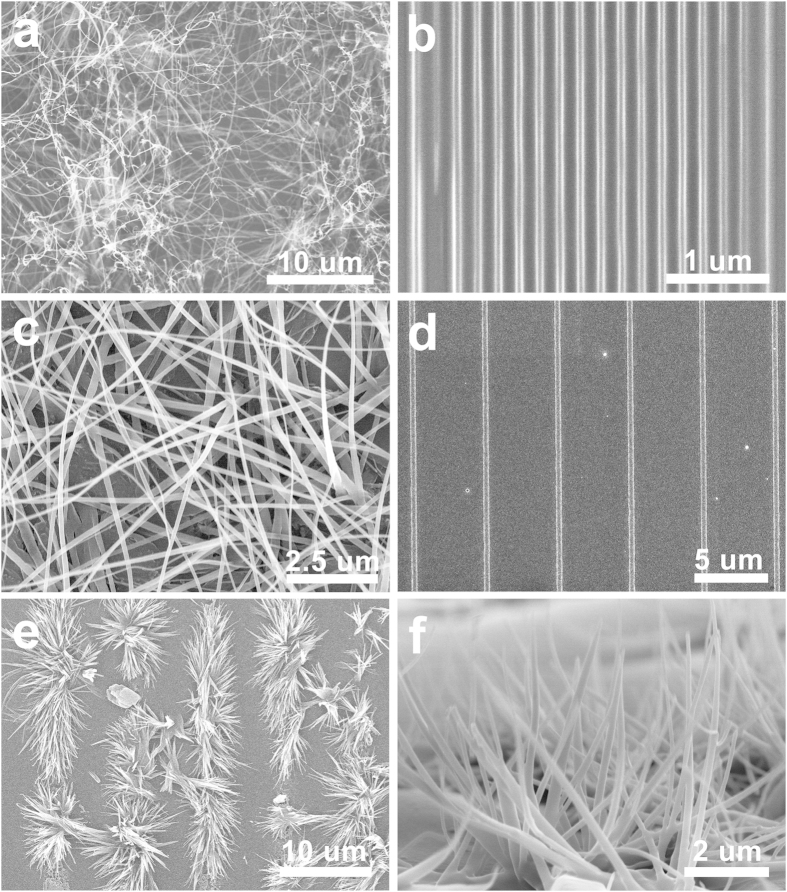
(**a**–**d**) Rubrene nanowires grown on the hydrophobically modified substrates. SEM micrographs of RB-NW_BA_ prepared by (**a**) eutectic melt crystallization, and (**b**) EMAN. The minimum feature size of the RB-NW_BA_ pattern was 80 nm. SEM micrographs of RB-NW_SA_ prepared by (**c**) eutectic melt crystallization, and (**d**) EMAN. (**e**–**f**) RB-NW_BA_ grown on hydrophilically modified substrate. Unlike RB-NW_BA_ grown on the hydrophobically modified substrates, RB-NW_BA_ grew vertically on the hydrophillically modified sustrate.

The preferential growth of rubrene on the (002) plane of BA during eutectic melt crystallization was utilized to control the nanowire growth in vertial or lateral direction on the substrate. The attachment energy calculation of benzoic acid reveals that the (002) facet is the only hydrophobic surface^44^,^45^, and other facets are hydrophilic. Especially, the (011) plane is the most hydrophilic facet of BA (Figure S3). We assumed that BA grows laterally or vertically depending on the surface energy of substrate: the hydrophobic (002) facet is preferentially laid down on the hydrophobically modified surface while the hydrophilic (011) facet is faced down to the hydrophilically modified surface to minimize interfacial energy. Since the growth of RB-NW_BA_ is guided by the (002) surface of BA, the nanowires can grow laterally on a hydrophobically modified surface and vertically on a hydrophilically modified surface. As shown in Fig. 5a,b, RB-NW_BA_ grew laterally on the hydrophobic silicon wafers modified with PDMS (water contact angle *θ *~ 107°). In contrast to this, vertically grown RB-NW_BA_ were dominantly observed on the hydrophilic silicon wafers modified with UV-O_3_ (water contact angle *θ *< 5°) (Fig. 5e,f). It is noteworthy that such apparent directional growth of rubrene nanowires was not observed when NAP was used as a matrix material instead of BA. This is presumably because the surface energy of the open facets of NAP crystal is similar to each other unlike benzoic acid. These all data suggest that the growth of RB-NW_BA_ is guided by the growth of benzoic acid.

### Preparation of rubrene nanowires using different VCAs

Since the growth of rubrene nanowires is guided by the VCA during the eutectic melt crystallization, the crystal structure and growth direction of rubrene nanowires can be further controlled by using different VCAs. To investigate this, rubrene nanowires were grown using different VCAs including SA and TCB. For SA, the eutectic temperature (*T*_e_ = 133 °C) was slightly higher than that of rubrene/BA mixture (*T*_e_ = 119 °C). For TCB, the eutectic temperature was only *T*_e_ = 40.8 °C. In this case, the process temperature window was accordingly adjusted depending on the eutectic temperature. Following the same processes outlined earlier, rubrene nanowires were prepared by eutectic melt crystallization using SA (RB-NW_SA_) and TCB (RB-NW_TCB_) as a VCA. As shown in Fig. 5c,d, the morphology of RB-NW_SA_ was very similar to that of RB-NW_BA_. However their crystal structures were different each other. The crystal structure analyzed by SAED and XRD (Fig. 4b(ii)) reveales that the RB-NW_SA_ is single-crystalline triclinic structure (*a* = 7.02, *b* = 8.54, *c* = 11.95, *α* = 93.04, *β* = 105.58, *γ* = 96.28)^36^. To understand the rubrene crystal growth on SA, the interface energy between salicylic acid and rubrene was calculated too. As summarized in Table 2 and Figure S4, the (010) plane of triclinic rubrene favorably grows on the (011) plane of SA along [100] direction. In this case, the *a*-axis of rubrene is aligned along the *a*-axis of SA. These calculation results are well consistent with the crystal structure of rubrene nanowires determined by SAED and XRD.

TCB was also used as a VCA for the eutectic melt crystallization of rubrene. As shwon in Fig. 4b(iii), XRD and SAED data showed that the resulting RB-NW_TCB_ was orthorhombic single crystal. It is notable that the crystal structure of RB-NW_TCB_ was same as that of RB-NW_BA_, but the growth direction was different each other. As summarized in Table 3 and Figure S5, RB-NW_TCB_ prefernentially grew along [010] direction while RB-NW_BA_ grew along [001] direction. These calculation results are also well consistent with the crystal structure of rubrene nanowires determined by SAED and XRD.

The eutectic melt crystallization is also highly applicable to many other organic semiconductors. As shown in Figure S6, Alq_3_, copper phthalocyanine and pentacene were crystallized by eutectic melt crystallization.

### Nanopatterning of organic semiconductors by eutectic-melt-assisted nanoimprinting (EMAN)

Since organic semiconductors can be melted at moderately low temperature by eutectic system, the conventional soft-lithography using PDMS mold was applicable to create nanopatterns of organic semiconductors (Fig. 1c). For example, to create nanopatterns of RB-NW_BA_, the powder mixtures of rubrene and BA was first placed on a substrate, and which was covered with a PDMS mask. The sample was then heated to melt the powder completely. At this time, molten liquid was sucked into the grooves of PDMS mold by the capillary force. The extra liquid was removed by soaking out using a paper. The sample was cooled down to room temperature. After removing the PDMS mold, the sample was placed in a vacuum to remove BA. As shown in Fig. 4b,d, RB-NW_BA_ and RB-NW_SA_ patterns were created by eutectic-melt-assisted nanoimprinting (EMAN). In this case, the minimum feature size of the pattern was 80 nm, and the length was on the order of several hundred micrometers. As shown in the previous section, RB-NW_BA_ can be vertically grown by modulating the surface energy of substrate. While the laterally grown RB-NW_BA_ patterns were observed on a hydrophobic substrate, RB-NW_BA_ were grown vertically when EMAN was conducted on a hydrophilic substrate (Fig. 5e,f).

### Electric properties of rubrene nanowires and their dependence on the crystallizable additive

**Figure 6:**
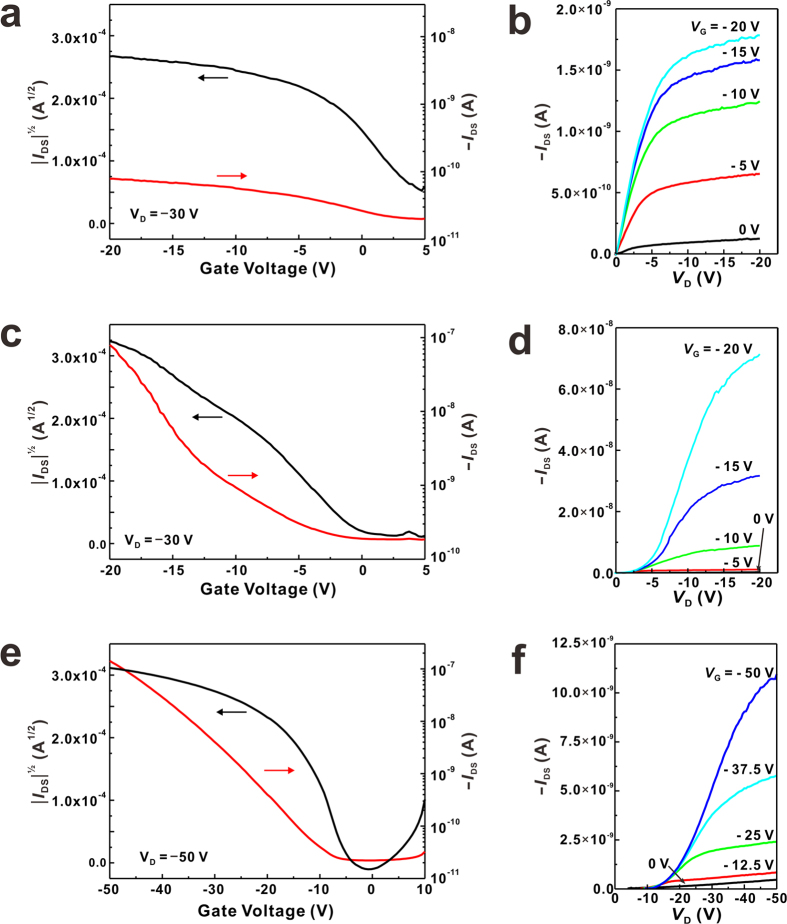
Representative transfer and output curves of OFET using (**a**,**b**) RB-NW_BA_, (**c**,**d**) RB-NW_TCB_, and (**e**,**f**) RB-NW_SA_.

**Figure 7:**
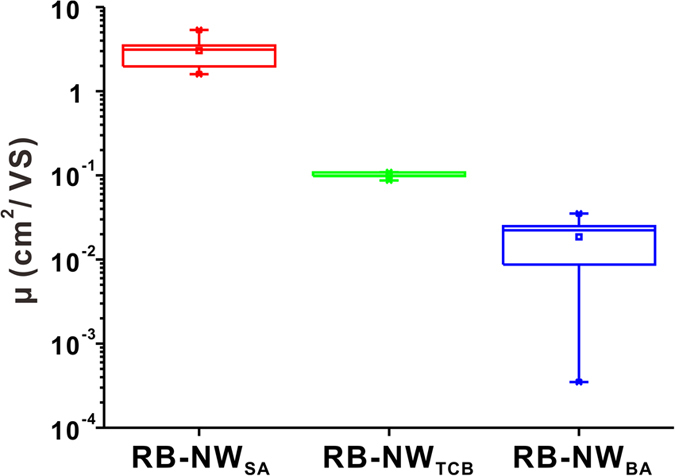
Average field effect hole mobility of RB-NW_BA_, RB-NW_TCB_, and RB-NW_SA_.

As demonstrated in the previous sections, the crystal structure and the growth direction of rubrene nanowires can be controlled by the species of VCA. Since the electric properties of materials are highly dependent on their both crystal structure and growth direction, it is expected that the rubrene nanowires prepared by using different VCAs exhibit different electric properties. To investigate this, the field-effect mobility of rubrene nanowires was measured. To fabricate the field-effect transistors, rubrene nanowire patterns with 100–300 nm width (RB-NW_BA_, RB-NW_SA_ and RB-NW_TCB_) were first created by EMAN, and then they were transferred on a highly doped p-type silicon wafer (100) with a thermally grown 200 nm thick oxide layer using a liquid-bridge-mediated nanotransfer moulding (LB-nTM) method^46^. By using LB-nTM, the laterally aligned rubrene nanowires can be transferred on the hydrophilic SiO_2_ substrate. Finally, 200 nm thick gold electrodes separated by 18 μm or 100 μm were vacuum deposited through a shadow mask on the rubrene nanowire patterns. Current-voltage (*I*—*V*) characteristics of the rubrene nanowires were measured using bottom-gate FET geometry under ambient condition at room temperature, and the saturation regime mobility was calculated. The channel width and length was measured to calculated the mobility from SEM images (Figure S7). The typical transfer and output characteristics of RB-NW_BA_, RB-NW_SA_ and RB-NW_TCB_ are shown in Fig. 6, and their average mobilities for 30 devices were shown in Fig. 7. The average mobility was 0.019, 0.10 and 3.05 cm^2^V^−1^ s^−1^ for RB-NW_BA_, RB-NW_TCB_ and RB-NW_SA_ respectively. The highest mobility was 0.035, 0.109 and 5.33 cm^2^V^−1^ s^−1^ for RB-NW_BA_, RB-NW_TCB_ and RB-NW_SA_ respectively. RB-NW_SA_ exhibiting the highest mobility has a triclinic structure with a growth direction along *a*-axis, while RB-NW_TCB_ and RB-NW_BA_ has a orthorhombic structure a the growth direction along *b*- and *c*-axis, respectively. These results are different from other previous reports showing that the orthorhombic rubrene typically shows higher mobility than that of the triclinic rubrene^47^,^48^. Since the mobility of organic semiconductors are highly dependent on the interfacial contact with the dielectric layer as well as electrodes^49^,^50^, residual impurities^51^,^52^, we are currently investigating the reversed mobility.

## Conclusions

In summary, we have successfully demonstrated the eutectic melt crystallization of various organic semiconductors. Due to the formation of eutectic system between organic semiconductors and VCAs, they can be melted and crystallizable at low temperature (*T*_e_ = 40.8 °C for rubrene/TCB). Due to the concurrent crystallization of semiconductor and VCA at the eutectic composition, the crystallization of semiconductor was guided by the crytallization of VCA. Threby the crystal structure and growth direction of semiconductor nanowires were controlled by using different VCAs. In this case, the resultant semiconductor nanowires were single crystalline. For example, RB-NW_BA_ and RB-NW_TCB_ formed the orthorhomic structure with growth direction of *c*- and *b*-axis, respectively while RB-NW_SA_ made the triclinic structure. Theoretical calculations showed that both interaction energy and cell distortion energy at the interface between rubrene and VCA affect crystal structure and growth direction. Due to the lowered melting temperature by eutectic system, the conventional soft-lithography technique was applicable to create well-defiend nanopatterns of organic semiconductors by eutectic melt crystallization. We have demonstrated that rubrene nanopattern with the minumum feature size of 80 nm can be created by EMAN. We anticipate that our eutectic melt crystallization technique provides a facile way of fabricating single crystalline nanodevices based on various organic semiconductors with the capability of controlling crystal structure, growth direction, patterning, and large scalability.

### Experimental and Computational Details

#### Materials and Instruments

Rubrene (99%) was purchased from TCI (Tokyo, Japan). All other chemicals were purchased from Sigma Aldrich. VCAs were used after purification by sublimation. All other chemicals were used as-purchased. The melting temperature was measured on TA Instruments SDT-Q600. Silicon wafers were purchased from LG Siltron (Kumi, Korea). SEM micrographs were taken on a Hitachi S-4800. TEM micrographs and SAED patterns were obtained on a Zeiss EM-912 OMEGA at 120 kV. XRD patterns were recorded using a Rigaku D/MAX-2000. MALDI-TOF spectra were taken on a Shimadzu biotech AXIMA. Electrical characterizations of the devices were measured on a semiconductor parameter analyzer (HP 4155 C, Agilent Technologies) in ambient condition at room temperature.

#### Surface Modification of Substrates

Hydrophobically or hydrophilically modified silicon wafers were used for the controlled growth of rubrene nanowires in vertical and lateral direction. For hydrophilic modification, silicon wafers were treated with UV-O_3_ for 10 min. After modification with UV-O_3_, the water contact angle of the wafer was measured at *θ* = 0°. For hydrophobic modification, silicon wafers were first treated with UV-O_3_, followed by immersing them into 0.5 wt% aqueous solution of 3-(aminopropyl triethoxysilane) (APTES) for 10 min. After washing with distilled water to remove residual APTES, wafers were reacted with monoglycidyl ether terminated PDMS (M_n_ = 5,000 g/mol) at 80 °C for 4 h, and then finally immersed into isopropyl alcohol with sonication for 1 min to remove the unreacted PDMS. After modification with PDMS, the water contact angle of the wafer was measured at *θ* = 107°.

#### Calculation of Attachment Energies

The calculation of attachment energy of organic crystals allow us both to study particle shape and to consider the effects of altering the growth rate of particular faces on crystal morphology. The attachment energy is calculated for a series of suitable slices (*hkl*). The calculated attachment energy is an average of the surfaces with Miler indices (*hkl*) and (−*h* −*k* −*l*). The ratio of the surface normal distances of all planes from the center of the polyhedron is determined according to the attachment energy. The final shape of the polyhedron is then determined by the intersection of the cleavage planes. The attachment energy, *E*_*att*_, is defined as the energy released on attachment of a growth slice to a growing crystal surface^41^. *E*_*att*_^42^ is computed as:


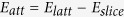


where *E*_*latt*_ = lattice energy of the crystal, *E*_*slice*_ = energy of a growth slice of thickness *d*_*hkl*_, and growth rate is proportional to *E*_*att*._ The growth rate of the crystal face is assumed to be proportional to its attachment energy; that is, faces with the lowest attachment energies are the slowest growing and, therefore, have the most morphological importance.

#### Calculation of Interface Energies

To understand the epitaxial growth of rubrene crystal on a VCA during the eutectic melt crystallization, the interface energy between a rubrene crystal and a solvent crystal was calculated. For the case of RB-NW_BA_, hydrophobic parts of BA molecule (benzene rings) face toward (002) surface, while hydrophilic parts of benzoic acid molecule (carboxyl groups) are mainly exposed to (010) surface, as shown in Figure S3. Although carboxyl groups can be exposed to (001) surface in the structural viewpoint, if we consider the strong hydrogen bond interaction between carboxyl groups, another benzoic acid molecule will cover the carboxyl group exposed surface (001). Consequentially, the growth morphology of BA has hydrophobic (002) surface instead hydrophilic (001) surface. Therefore, we considered the interface interaction between the hydrophobic (002) surface of BA and the surfaces of rubrene, (100), (010) and (001). We also considered rubrene growth direction because BA has needle-like crystal structure. In order to investigate the stability of interfaces between rubrene and BA, we calculated cell distortion energy (*E*_*cd*_) and interaction energy (*E*_*ie*_) using the constructed rubrene/BA interface structures. Because of structural mismatching, growing rubrene crystal feels stress at the interfaces. The average structural deviation of rubrene structure is 5.52–12.75% depending on the facet of rubrene. Hence, the *E*_*cd*_ is defined the energy difference of rubrene surface structures, original structure and distorted structure with BA cell parameters, and the *E*_*ie*_ is interaction energy between rubrene surface with (002) BA surface at relaxed interface structure. Therefore, we can evaluate the stability of interfaces between rubrene and BA with interface formation energy (*E*_*f*_), which is sum of *E*_*cd*_ and *E*_*ie*_. (100) and (001) surfaces show relatively large *E*_*cd*_ values compared to (010) surface. In contrast, looking at the *E*_*ie*_ values, all rubrene surfaces give attractive interaction with (002) BA surface regardless of facet type and growth direction. Especially, (001) surface of rubrene has the strongest interaction energy. Considering two effects, only (010) rubrene surface can create energetically stable rubrene/BA interface with exothermic interface formation energy (*E*_*f*_). It is also seen that the growth direction prefers *c*-axis even though the energy difference is not so large compared to *a*-axis. The favorable facet and growth direction of rubrene on the (002) BA were determined by calculating both the cell distortion energy and the interaction energy. Considering both interaction energy and cell distortion energy between rubrene surfaces and (002) BA, it is seen that the interface is most stabilized when the (010) rubrene surface along the *c*-axis grown on the (002) facet of BA along the *b*-axis, as shown in Figure S3. This calculation results are well consistent with the crystal structure of rubrene nanowires determined by XRD and SAED. All these data suggest that rubrene nanowire growth by eutectic solidification is guided by the growth of BA. Similarly, the interface energies between rubrene/SA and rubrene/TCB were calculated (Tables 2 and 3, Figure S4 and 5).

## Additional Information

**How to cite this article**: Chung, J. *et al*. Controlled Growth of Rubrene Nanowires by Eutectic Melt Crystallization. *Sci. Rep*. **6**, 23108; doi: 10.1038/srep23108 (2016).

## Supplementary Material

Supplementary Information

## Figures and Tables

**Table 1:** Calculation results of interaction energy and cell distortion energy between (002) surface of BA and several different rubrene surfaces.

Surface	Growth Direction	Interaction Energy with BA Surface (002) (E_ie_, kcal/mol)	Cell Distortion Energy (E_cd_, kcal/mol)	Interface Formation Energy (E_ie_ + E_cd_)
**(100)**	c-axis	−7.06	11.55	4.49
	b-axis	−7.22	11.55	4.33
**(010)**	a-axis	−3.29	2.53	−0.76
	**c-axis**	**−3.34**	**2.53**	−**0.81**
**(001)**	b-axis	−17.67	19.06	1.39
	a-axis	−17.36	19.06	1.70

**Table 2:** Calculation results of interaction energy and cell distortion energy between (011) surface of SA and several different rubrene surfaces.

Surface	Growth Direction	Interaction Energy with SA (011) (E_ie_, kcal/mol)	Cell Distortion Energy (E_cd_, kcal/mol)	Interface Formation Energy (E_ie_ + E_cd_)
**(100)**	b-axis	−4.94	4.71	−0.23
**(010)**	**a-axis**	**−1.79**	**−0.42**	−**2.21**
**(001)**	b-axis	−1.94	1.61	−0.33

**Table 3:** Calculation results of interaction energy and cell distortion energy between (011) surface of TCB and several different rubrene surfaces.

Surface	Growth Direction	Interaction Energy with TCB (011) (E_ie_, kcal/mol)	Cell Distortion Energy (E_cd_, kcal/mol)	Interface Formation Energy (E_ie_ + E_cd_)
**(100)**	b-axis	6.63	−3.58	3.05
**(010)**	c-axis	−2.32	3.16	0.84
**(001)**	**b-axis**	**3.17**	−**8.38**	−**5.21**
