# Supplementary material for: Controlled Growth of Rubrene Nanowires by Eutectic Melt Crystallization
Source: Sci Rep. 2016 Mar 15;6:23108. doi: 10.1038/srep23108 (PMC4791557; doi:10.1038/srep23108)
Supplement: Supplementary Information [file srep23108-s1.pdf]

(In Preparation as an *Aricle* to the Editor of *Scientific Reports*)

Supporting Information

Controlled Growth of Rubrene Nanowires by Eutectic Melt Crystallization

Jeyon Chung[1†], Jinho Hyon[1†], Kyung-Sun Park[1], Boram Cho[1], Jangmi Baek[1], Jueun Kim[1], Sang Uck Lee[2], Myung Mo Sung[1], Youngjong Kang[1]*

[1] Department of Chemistry, Research Institute for Natural Sciences, and Institute of Nano Science and Technology, Hanyang University, 222 Wangsimni-Ro, Seongdong-Gu, Seoul, 04763 (Korea)
[2] Department of Chemistry and Applied Chemistry, Hanyang University, 55 Hanyangdaehak-ro, Sangnok-gu, Ansan, Gyeonggi-do, 15588 (Korea)

* e-mail: youngjkang@hanyang.ac.kr
[†] These authors contributed equally to this work.

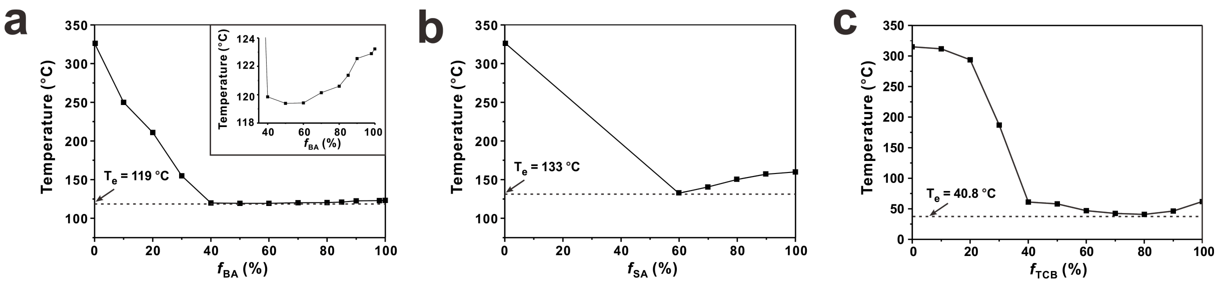

Figure S1. The change of melting point for the mixtures of a) rubrene and BA, b) rubrene and SA, and c) rubrene and TCB as a function of the weight fraction of a crystallizable solvnet (f_X). Melting temperatures were determined by using DSC.

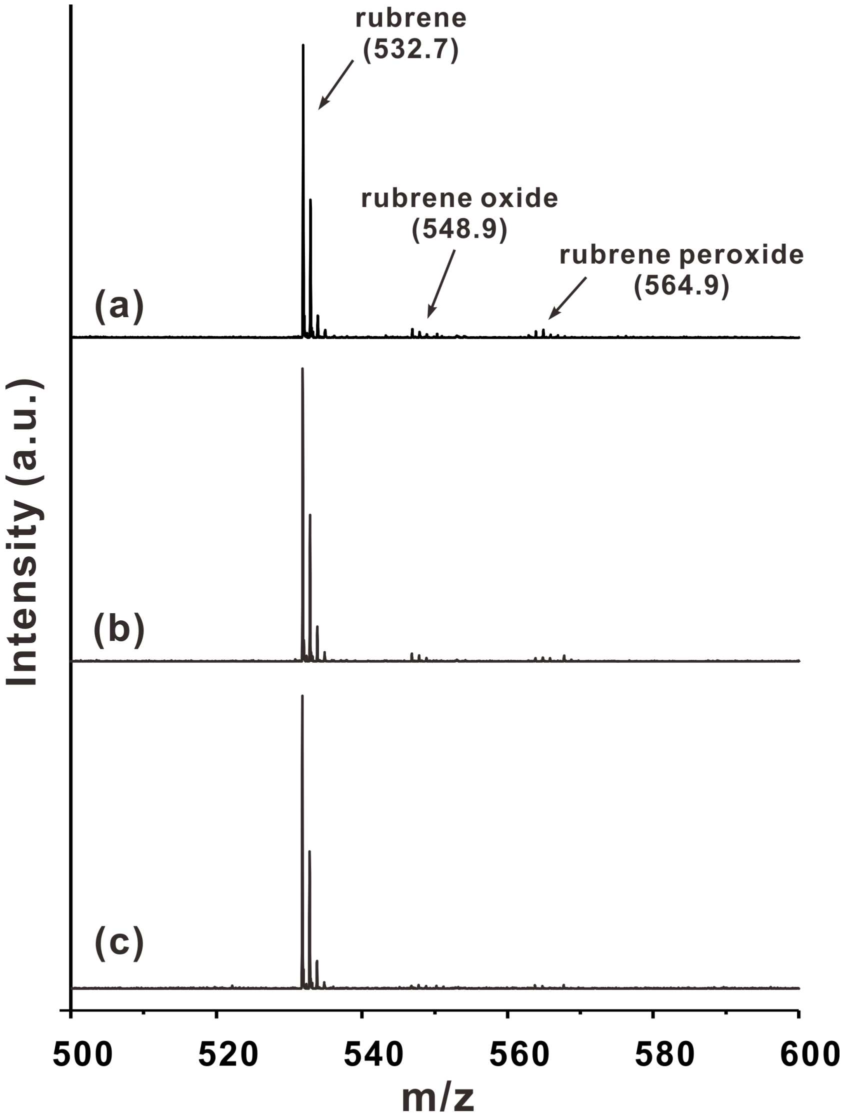

Figure S2. MALDI spectra of a) rubrene powder as purchased, b) rubrene powder annealed at 130 °C for 24 hours, and c) rubrene-NW_{BA} prepared by eutectic melt crystallization. Each peak is assigned as pure rubrene ($C_{42}H_{28}$, m/z = 532.7 g/mol), rubrene endooxide ($C_{42}H_{28}O$, m/z =548.9 g/mol), and rubrene endoperoxide ($C_{42}H_{28}O_2$, m/z = 564.9 g/mol). The relative contents of rubrene endooxide and rubrene endoperoxide were measured at a) 1.1% and 2.6%, b) 1.1% and 1.3%, and c) 0.1% and 0.2%, and respectively.

Rubrene is known to be easily oxidized in the ambient condition. Chemical analysis of naturally oxidized rubrene crystal shows that the relative concentration of rubrene peroxide can be as much as several percentages, and which makes significant modification of electronic characteristics.[1-4] To characterize the stability of rubrene during the eutectic melt crystallization, the contents of rubrene oxide ($C_{42}H_{28}O$) and rubrene peroxide ($C_{42}H_{28}O_2$) were analyzed by means of MALDI-TOF spectrometry. As shown in Figure S2, the as-purchased rubrene powder was slightly oxidized exhibiting rubrene oxide peak at m/z = 548.9 (1.1%) and rubrene peroxide peak at m/z =564.9 (2.6%). Interestingly, the contents of oxidized rubrene

significantly decreased after eutectic melt crystallization (0.1% of rubrene oxide and 0.21% of rubrene peroxide) (Figure S2c). It is known that rubrene peroxide has a relatively low activation energy for thermal decomposition (E_a = 30.4 kcal/mol), so that rubrene oxide can be decomposed to rubrene at slightly elevated temperature (130 °C).[1] While thermal decomposition of rubrene peroxide was also observed when neat rubrene power was annealed at 130 °C (Figure S2b), the residue of rubrene oxide (1.1%) and rubrene peroxide (1.3%) was higher than that of the sample prepared by eutectic melt crystallization.

<Theoretical Calculations of Rubrene Nanowires on Various VCAs>

To determine how rubrene crystals grow with VCAs during the eutectic crystallization, the interaction energy and the cell distortion energy between rubrene and VCAs were calculated. The crystal morphologies and facets of orthorhombic/triclinic rubrene and VCAs (BA, SA and TCB) were first calculated by using the attachment energy theory.[5-7] In this case, the main facets of VCAs for the growth of rubrene were determined as (002) for BA, (011) for SA, and (011) for TCB respectively by considering their surface energy (Figure S3a, 4a, 5a). The growth facets and growth directions of rubrene on VCAs were determined by calculating the interaction and the cell distortion energy at the inferfaces of rubrene and VCAs. The calculations were summaried in Table 1-3 for BA, SA and TCB respectively. The growth directions and interfacial facets of rubrene were determined by minimizing the sum of the interaction and cell distortion energy. The resultant geometries of rubrene and VCSs were depicted in Figure S3b, 4b and 5b for RB-NW_{BA}, RB-NW_{SA}, and RB-NW_{TCB} respectively.

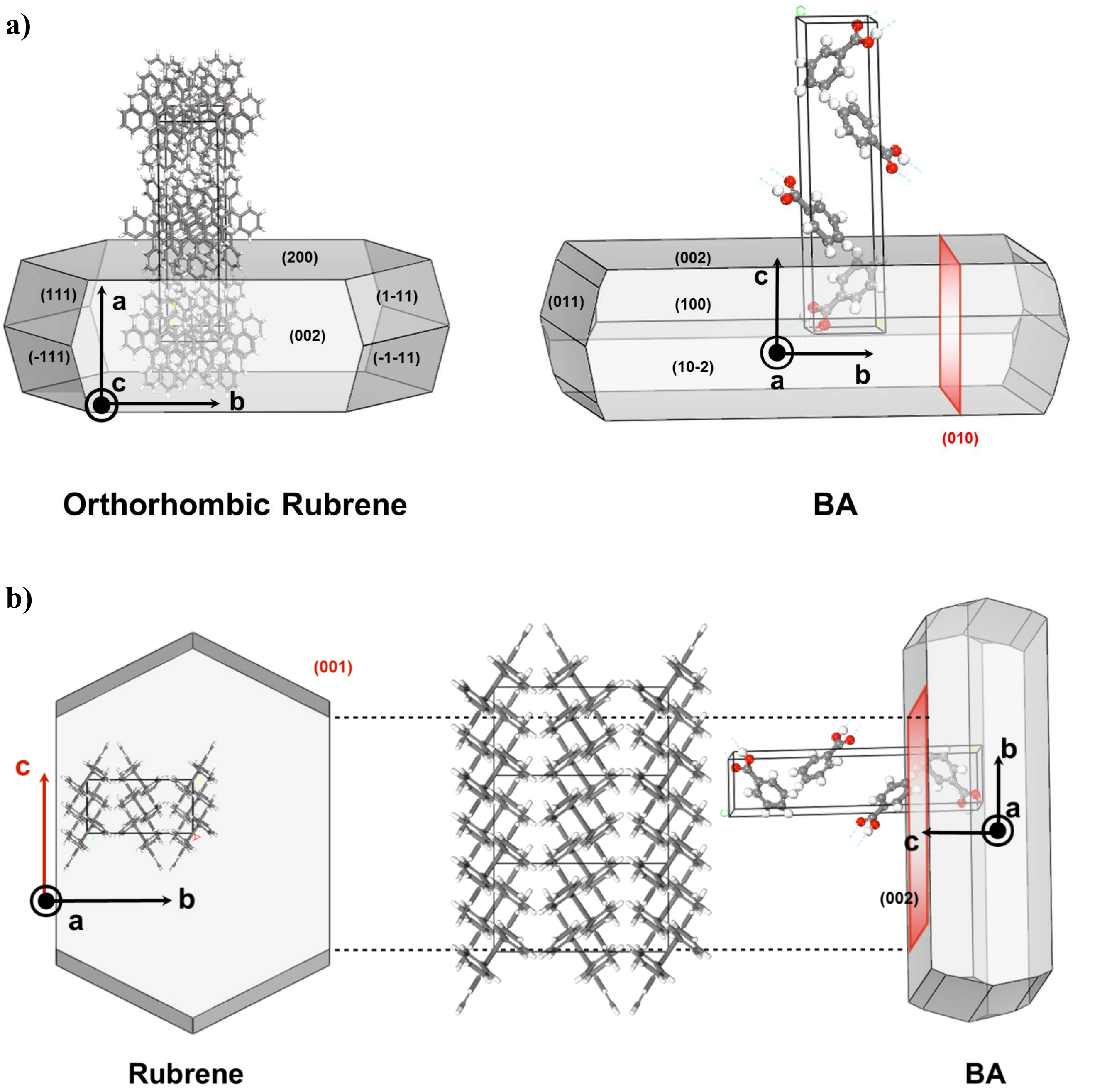

Figure S3. a) The crystal structure of orthorhombic rubrene and benzoic acid. b) Growth of orthorhombic rubrene crystal on the (002) benzoic acid surface along with *c*-axis direction.

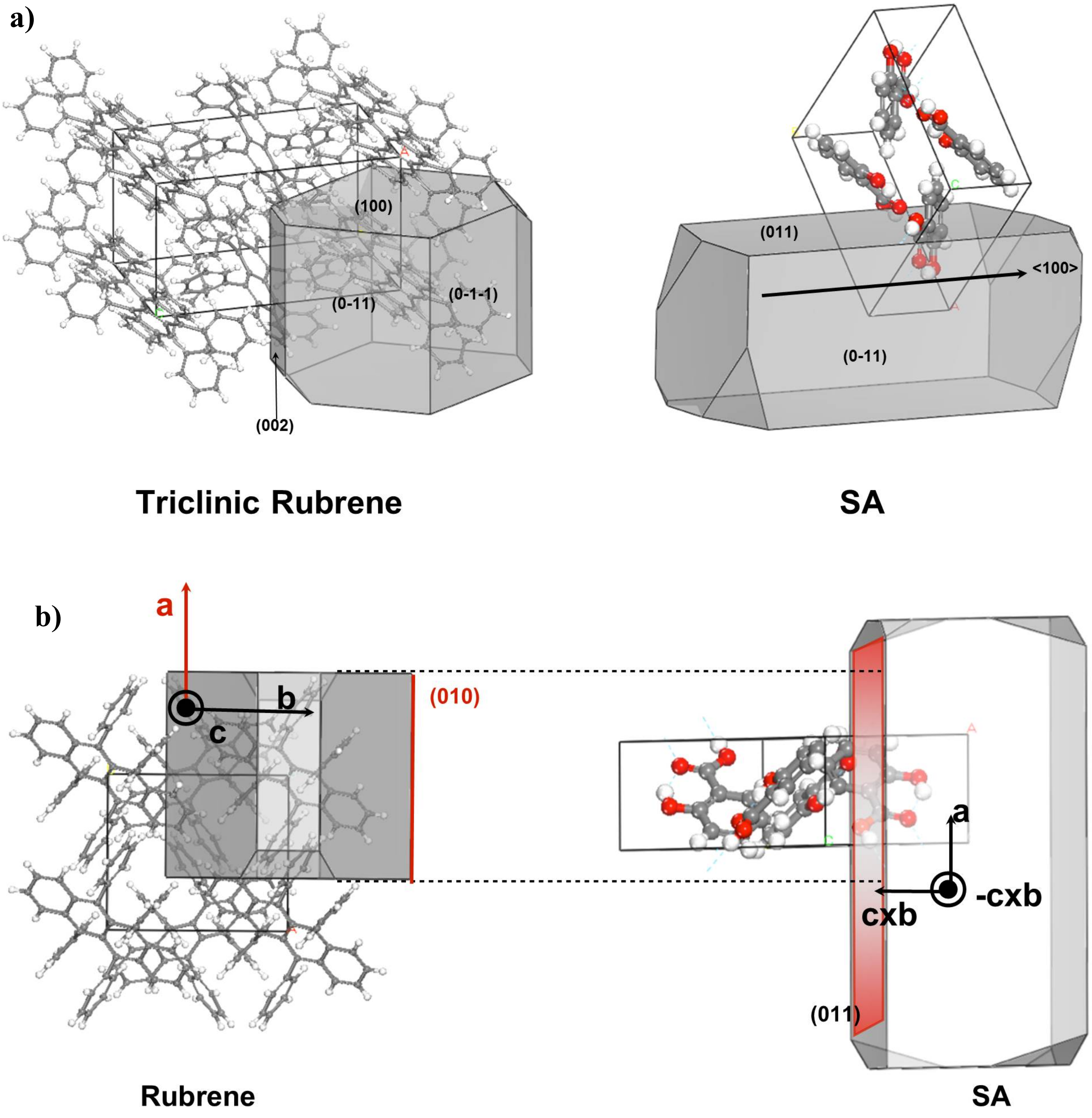

Figure S4. a) The crystal structure of triclinic rubrene and salicylic acid. b) Growth of triclinic rubrene crystal on the (011) salicylic acid surface along with *a*-axis direction.

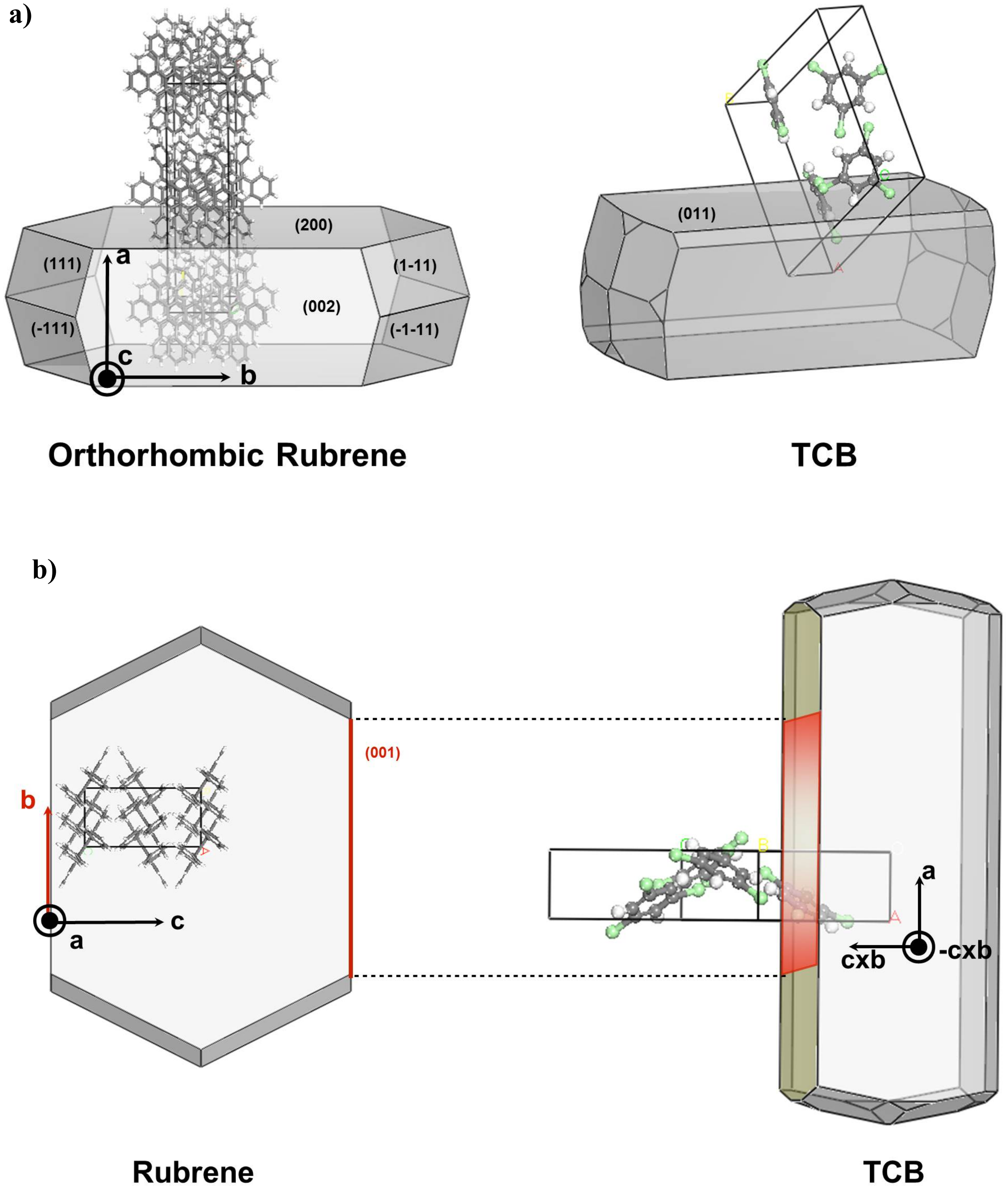

Figure S5. a) The crystal structure of orthorhombic rubrene and 1,3,5-trichlorobenzene. b) Growth of orthorhombic rubrene crystal on the (011) 1,3,5-trichlorobenzene surface along with *b*-axis direction.

<Eutectic Melt Crystallization of Various Organic Semiconductors>

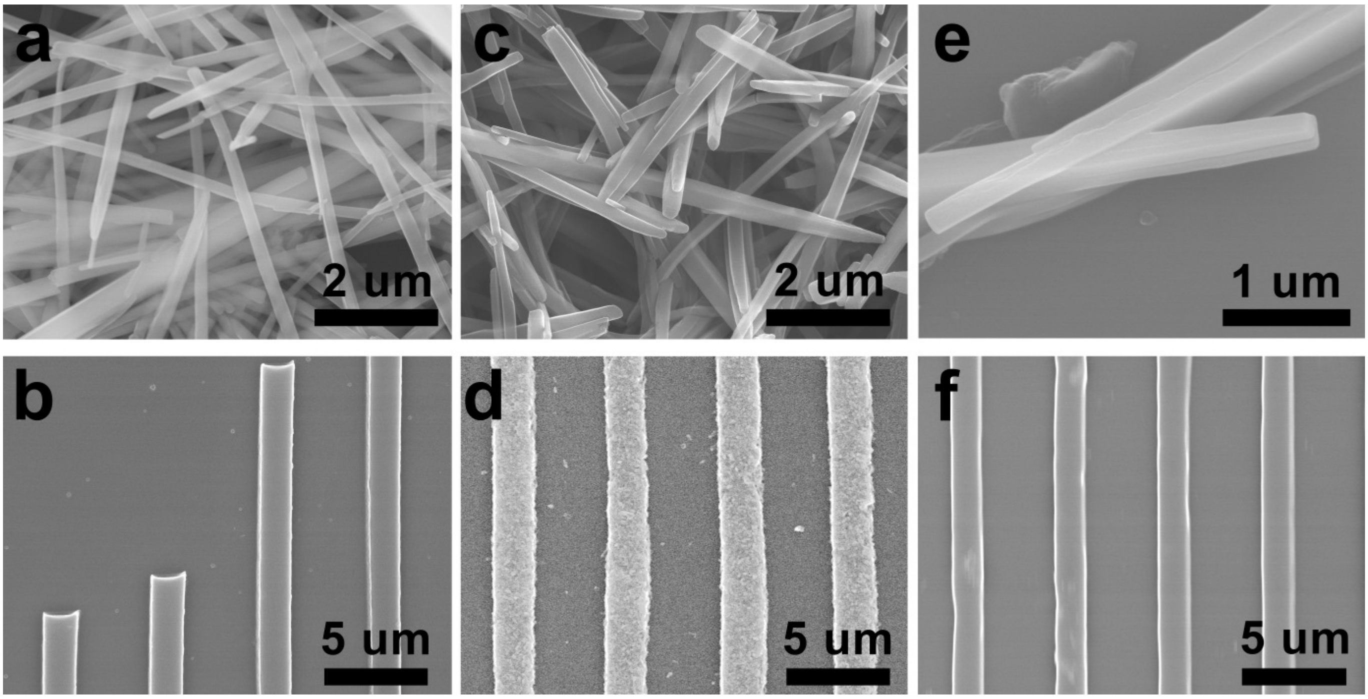

Figure S6. The crystal growth of various organic semiconductors using eutectic melt crystallization. Crystals were prepared by using the mixture of (a, b) Alq_3 and NAP, (c, d) copper phthalocyanine and NAP, and (e, f) pentacene and TCB.

Our eutectic melt crystallization method is highly applicable to many other organic semiconductors including Alq_3, copper phthalocyanine and pentacene. The general procedures and compositions are almost same as those described in the main body for rubrene. NAP was used as a matrix for the growth of Alq_3 and copper phthalocyanine, and TCB was chosen for the growth of pentacene. Remarkably pentacene which is insoluble in most organic solvent can be grown by eutectic reaction with TCB at moderate temperature (~60 °C).

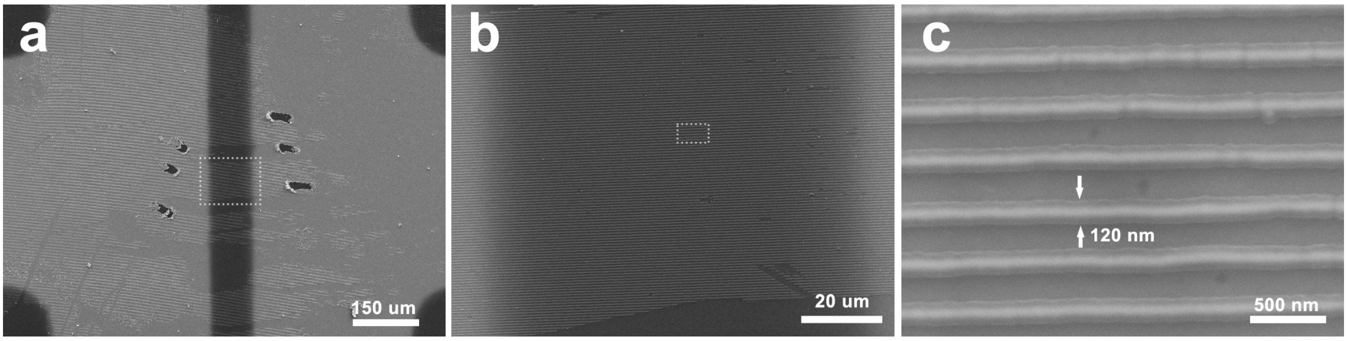

Figure S7. Representative SEM images of FET device for mobility measurement. Image (b) and (c) is the magnified image of the indicated area in image (a) and (b) respectively.

References

1 Hochstrasser, R. M. & Ritchie, M. The Photoformation and Thermal Decomposition of Rubrene Peroxide. *Trans. Faraday Soc.* **52**, 1363-1373 (1956).

2 Mitrofanov, O. *et al.* Oxygen-Related Band Gap State in Single Crystal Rubrene. *Phys. Rev. Lett.* **97**, 166601 (2006).

3 Podzorov, V., Pudalov, V. & Gershenson, M. Light-Induced Switching in Back-Gated Organic Transistors with Built-in Conduction Channel. *Appl. Phys. Lett.* **85**, 6039-6041 (2004).

4 Takahashi, T., Takenobu, T., Takeya, J. & Iwasa, Y. Ambipolar Organic Field-Effect Transistors Based on Rubrene Single Crystals. *Appl. Phys. Lett.* **88**, 033505 (2006).

5 Hartman, P. The Attachment Energy as a Habit Controlling Factor Ii. Application to Anthracene, Tin Tetraiodide and Orthorhombic Sulphur. *J. Cryst. Growth* **49**, 157-165 (1980).

6 Hartman, P. & Bennema, P. The Attachment Energy as a Habit Controlling Factor: I. Theoretical Considerations. *J. Cryst. Growth* **49**, 145-156 (1980).

7 Docherty, R., Clydesdale, G., Roberts, K. & Bennema, P. Application of Bravais-Friedel-Donnay-Harker, Attachment Energy and Ising Models to Predicting and Understanding the Morphology of Molecular Crystals. *J. Phys. D: Appl. Phys.* **24**, 89-99 (1991).
